# Exact topological inference of the resting-state brain networks in twins

**DOI:** 10.1162/netn_a_00091

**Published:** 2019-07-01

**Authors:** Moo K. Chung, Hyekyoung Lee, Alex DiChristofano, Hernando Ombao, Victor Solo

**Affiliations:** University of Wisconsin, Madison, WI, USA; Seoul National University, Seoul, Korea; Washington University, St. Louis, USA; King Abdullah University of Science and Technology, Thuwal, Saudi Arabia; University of New South Wales, Sydney, Australia

**Keywords:** Functional brain networks, Cycles, Betti numbers, Persistent homology, Twin imaging studies, Resting-state fMRI

## Abstract

A cycle in a brain network is a subset of a connected component with redundant additional connections. If there are many cycles in a connected component, the connected component is more densely connected. Whereas the number of connected components represents the integration of the brain network, the number of cycles represents how strong the integration is. However, it is unclear how to perform statistical inference on the number of cycles in the brain network. In this study, we present a new statistical inference framework for determining the significance of the number of cycles through the Kolmogorov-Smirnov (KS) distance, which was recently introduced to measure the similarity between networks across different filtration values by using the zeroth Betti number. In this paper, we show how to extend the method to the first Betti number, which measures the number of cycles. The performance analysis was conducted using the random network simulations with ground truths. By using a twin imaging study, which provides biological ground truth, the methods are applied in determining if the number of cycles is a statistically significant heritable network feature in the resting-state functional connectivity in 217 twins obtained from the Human Connectome Project. The MATLAB codes as well as the connectivity matrices used in generating results are provided at http://www.stat.wisc.edu/∼mchung/TDA.

## Author Summary

In this paper, we propose a new topological distance based on the Kolmogorov-Smirnov (KS) distance that is adapted for brain networks, and compare them against other topological network distances including the Gromov-Hausdorff (GH) distances. KS-distance is recently introduced to measure the similarity between networks across different filtration values by using the zeroth Betti number, which measures the number of connected components. In this paper, we show how to extend the method to the first Betti number, which measures the number of cycles. The performance analysis was conducted using random network simulations with ground truths. Using a twin imaging study, which provides biological ground truth (of network differences), we demonstrate that the KS distances on the zeroth and first Betti numbers have the ability to determine heritability.

## INTRODUCTION

The modular structure and connected components are the fundamental topological features of a brain network. Brain networks with a higher number of connected components have many disjointed clusters, and the transfer of information will likely be impeded. Modular structures are often studied through the Q-modularity in graph theory (Meunier, Lambiotte, Fornito, Ersche, & Bullmore, 2009; Newman, Barabasi, & Watts, 2006) and the zeroth Betti number in persistent homology (Carlsson & Memoli, 2008; Carlsson & Mémoli, 2010; Chung, Vilalta-Gil, Lee, Rathouz, Lahey, & Zald, 2017b; Chung, Luo, Leow, Adluru, Alexander, Richard, & Goldsmith, 2018b; Lee, Chung, & Lee, 2014).

Persistent homology provides a coherent framework for obtaining higher order topological features beyond modular structures (Edelsbrunner & Harer, 2008; Zomorodian & Carlsson, 2005). A brain network can be treated as the 1-skeleton of a simplicial complex, where the 0-dimensional hole is the connected component, and the 1-dimensional hole is a cycle. The number of *k*-dimensional holes is called the *k*-th Betti number and denoted as *β*_*k*_ (Lee et al., 2014; Lee, Chung, Kang, Choi, Kim, & Lee, 2018; Petri, Expert, Turkheimer, Carhart-Harris, Nutt, Hellyer, & Vaccarino, 2014; Sizemore, Giusti, Kahn, Vettel, Betzel, & Bassett, 2018). In this study, we will study higher order topological changes of brain networks using cycles. The cycle structure in networks is important for information propagation, redundancy, and feedback loops (Lind, Gonzalez, & Herrmann, 2005). If a cycle exists in the network, information can be delivered using two different redundant paths and interpreted as redundant connections. Alternately, it can be viewed as diffusing the spread of information and creating information bottlenecks (Tarjan, 1972).

Although cycles in a network have been widely studied in graph theory, especially in path analysis, they are rarely used in brain network analysis (Sporns, 2003; Sporns, Tononi, & Edelman, 2000). Existing graph analysis packages such as Brain Connectivity (http://sites.google.com/site/bctnet) do not provide any tools related to cycles. Traditionally, cycles are often computed using the brute-force depth-first search algorithm (Tarjan, 1972). In standard graph theoretic approaches, graph theory features are measured mainly by determining the difference in graph theory features such as assortativity, betweenness centrality, small-worldness, and network homogeneity (Bullmore & Sporns, 2009; Rubinov & Sporns, 2010; Rubinov, Knock, Stam, Micheloyannis, Harris, Williams, & Breakspear, 2009; Uddin, Kelly, Biswal, Margulies, Shehzad, Shaw, Ghaffari, Rotrosen, Adler, Castellanos, & Milham, 2008). Comparison of graph theory features appears to reveal changes of structural or functional connectivity associated with different clinical populations (Rubinov & Sporns, 2010). Since weighted brain networks are difficult to interpret and visualize, they are often turned into binary networks by thresholding edge weights (He, Chen, & Evans, 2008; Wijk, Stam, & Daffertshofer, 2010). However, the thresholds for the edge weights are often chosen arbitrarily and produce results that could alter the network topology and thus make comparisons difficult. To obtain the proper optimal threshold where comparisons can be made, the multiple comparison correction over every possible edge has been proposed (Rubinov et al., 2009; Wijk et al., 2010). However, the resulting binary graph is extremely sensitive depending on the chosen *p* value or threshold value. Others tried to control the sparsity of edges in the network in obtaining the binary network (Achard & Bullmore, 2007; Bassett, 2006; He et al., 2008; Lee, Kang, Chung, Kim, & Lee, 2012; Wijk et al., 2010). However, one encounters the problem of thresholding sparse parameters. Thus existing methods for binarizing weighted networks cannot escape the inherent problem of arbitrary thresholding.

There is currently no widely accepted criteria for thresholding networks. Instead of trying to find an optimal threshold that gives rise to a single network that may not be suitable for comparing clinical populations, cognitive conditions, or different studies, *why not use each network produced from every threshold?* Motivated by this simple question, a new multiscale hierarchical network modeling framework based on persistent homology has been proposed (Cassidy, Rae, & Solo, 2015; Chung, Hanson, Lee, Adluru, Alexander, Davidson, & Pollak, 2013; Giusti, Pastalkova, Curto, & Itskov, 2015; Lee, Chung, Kang, Kim, & Lee, 2011a, 2011b; Lee et al., 2012; Petri, Scolamiero, Donato, & Vaccarino, 2013; Petri et al., 2014; Sizemore, Giusti, & Bassett, 2016; Sizemore et al., 2018; Stolz, Harrington, & Porter, 2017). Persistent homology, a branch of computational topology (Carlsson & Memoli, 2008; Edelsbrunner & Harer, 2008; Edelsbrunner, Letscher, & Zomorodian, 2000), provides a more coherent mathematical framework for measuring network distance than the conventional method of simply taking the difference between graph theoretic features or the norm of the connectivity matrices. Instead of looking at networks at a fixed scale, as is usually done in many standard brain network analysis, persistent homology observes the changes of topological features of the network over multiple resolutions and scales (Edelsbrunner & Harer, 2008; Horak, Maletić, & Rajković, 2009; Zomorodian & Carlsson, 2005). In doing so, it reveals the most persistent topological features that are robust under noise perturbations. This robustness in performance under different scales is needed for most network distances that are parameter and scale dependent.

In persistent homology–based brain network analysis, instead of analyzing networks at one fixed threshold that may not be optimal, we build the collection of nested networks over every possible threshold by using the *graph filtration*, a persistent homological construct (Chung et al., 2013; Lee et al., 2011a, 2012). The graph filtration is a threshold-free framework for analyzing a family of graphs but requires hierarchically building specific nested subgraph structures. The graph filtration shares similarities to the existing multithresholding or multiresolution network models that use many different arbitrary thresholds or scales (Achard, Salvador, Whitcher, Suckling, & Bullmore, 2006; He et al., 2008; Kim, Adluru, Chung, Okonkwo, Johnson, Bendlin, & Singh, 2015; Lee et al., 2012; Supekar, Menon, Rubin, Musen, & Greicius, 2008). Such approaches are mainly used to visually display the dynamic pattern of how graph theoretic features change over different thresholds, and the pattern of change is rarely quantified. Persistent homology can be used to quantify such dynamic patterns in a more coherent mathematical framework. Recently, various persistent homological network approaches have been proposed. In Giusti et al. (2015) and Sizemore et al. (2016, 2018), graph filtration was developed on cliques. In Petri et al. (2013), weighted clique rank homology was developed. In Petri et al. (2014), the concept of homological scaffolds was developed and applied to the resting-state fMRI.

In persistent homology, there are various metrics that have been proposed to measure similarity and distances, including the bottleneck, Gromov-Hausdorff (GH), and Wasserstein distances (Chazal, Cohen-Steiner, Guibas, Mémoli, & Oudot, 2009; Kerber, Morozov, & Nigmetov, 2017; Tuzhilin, 2016), the complex vector method (Di Fabio & Ferri, 2015), and the persistence kernel (Ibanez-Marcelo, Campioni, Manzoni, Santarcangelo, & Petri, 2018a; Ibanez-Marcelo, Campioni, Phinyomark, Petri, & Santarcangelo, 2018b; Kusano, Hiraoka, & Fukumizu, 2016). Among them, the bottleneck and GH distances are possibly the two most popular distances that were originally used to measure distance between two metric spaces (Tuzhilin, 2016). They were later adapted to measure distances in persistent homology, dendrograms (Carlsson & Memoli, 2008; Carlsson & Mémoli, 2010; Chazal et al., 2009), and brain networks (Lee et al., 2011b, 2012). The probability distributions of bottleneck and GH-distances are unknown. Thus, the statistical inference on them can only be done through resampling techniques such as permutations (Lee et al., 2012; Lee, Kang, Chung, Lim, Kim, & Lee, 2017), which often cause serious computational bottlenecks for large-scale networks.

To bypass the computational bottleneck associated with resampling large-scale networks, the Kolmogorov-Smirnov (KS) distance was introduced (Chung et al., 2013, 1; Lee et al., 2017). The advantage of using KS-distance is that its gives results that are easier to interpret than those obtained from less intuitive distances from persistent homology. Furthermore because of its simplicity in construction, it is possible to determine its probability distribution exactly without resampling (Chung et al., 2017b). However, the KS-distance has been only applied to the number of connected components *β*_0_, and it is unclear how to apply to the number of cycles *β*_1_ in graphs and networks. In this paper, for the first time, we show how to extend the KS-distance by performing statistical inference on *β*_1_. This is achieved by establishing the monotonic property of the number of cycles over graph filtration. The monotonicity is then used in constructing the KS-distance for topologically differentiating two networks. Subsequently, the method is applied to the large-scale resting-state twin fMRI study in determining the heritability of the number of cycles.

## CORRELATION BRAIN NETWORK

The edge weight, which measures the strength of a connection, is usually given by a similarity measure between the observed data on the nodes in brain networks. Various similarity measures have been proposed. The correlation or mutual information between measurements for the biological or metabolic network and the frequency of contact between actors for the social network have been used as edge weights (Bassett, Meyer-Lindenberg, Achard, Duke, & Bullmore, 2006; Bien & Tibshirani, 2011; Li, Liu, Li, Qin, Li, Yu, & Jiang, 2009; McIntosh & Gonzalez-Lima, 1994; Newman & Watts, 1999; Song, Havlin, & Makse, 2005). In particular, the Pearson correlation has been most widely used as edge weights in functional brain network modeling.

Consider a weighted graph with node set *V* = {1, …, *p*} and edge weights *w* = (*w*_*ij*_) between nodes *i* and *j*. Let **x**_*j*_ = (*x*_1*j*_, ⋯, *x*_*nj*_)^⊤^ ∈ ℝ^*n*^ be *n* × 1 measurement vector on node *j*. Let us center and normalize data **x**_*j*_ such that

$${∥x_{j}∥}^{2}=x_{j}^{⊤}x_{j}=\overset{n}{\underset{i=1}{\sum }}x_{ij}^{2}=1,\;\overset{n}{\underset{i=1}{\sum }}x_{ij}=0.$$

Then we can show that *ρ*_*ij*_ = $x_{i}^{⊤}$**x**_*j*_ is the Pearson correlation between **x**_*i*_ and **x**_*j*_ (Chung, Hanson, Ye, Davidson, & Pollak, 2015). Note that correlations are invariant under scale and translations. Naturally, we are interested in using correlations or their simple functions such as

$$\rho_{ij}=x_{i}^{⊤}x_{j}\;\text{or}\;\rho_{ij}=1-x_{i}^{⊤}x_{j}$$

as edge weights. Among possible functions of correlations,

$$w_{ij}={\left(1-\rho_{ij}\right)}^{1/2}$$ (1)

satisfies triangle inequality *w*_*ij*_ ≤ *w*_*ik*_ + *w*_*kj*_ and other metric properties (Chung, Lee, Solo, Davidson, & Pollak, 2017a). Having metric distances facilitates more mathematically coherent interpretation of brain networks and offers many nice mathematical properties. With such edge weight *w*, 𝒳 = (*V*, *w*) forms a metric space. In the simulation studies in this paper, Equation 1 is used as the edge weights.

## GRAPH FILTRATION

All topological network distances that will be introduced in later sections are based on filtrations on graphs by thresholding edge weights.

Definition 1 Given weighted network 𝒳 = (*V*, *w*) with positive edge weight *w* = (*w*_*ij*_), the binary network 𝒳_*ϵ*_ = (*V*, *w*_*ϵ*_) is a graph consisting of the node set *V* and the binary edge weights *w*_*ϵ*_ given by

$$w_{\varepsilon }=(w_{ij,\varepsilon })=\left\{\begin{array}{ll} 1 & \text{if}\;w_{ij}>\varepsilon ;\\ 0 & \text{otherwise}. \end{array}\right.$$

Any edge weight less than or equal to *ϵ* is made into zero while edge weights larger than *ϵ* are made into one. Lee et al. (2011b, 1) defines the binary graphs by thresholding above, that is, *w*_*ij*,*ϵ*_ = 1 if *w*_*ij*_ <= *ϵ*, which is consistent with the definition of the Rips filtration. However, in brain imaging, the higher value of *w*_*ij*_ indicates stronger connectivity. Thus, we are thresholding below and leave out stronger connections (Chung et al., 2013, 1).

Note *w*_*ϵ*_ is the adjacency matrix of 𝒳_*ϵ*_, which is a simplicial complex consisting of 0-simplices (nodes) and 1-simplices (edges) (Ghrist, 2008). By increasing the filtration value *ϵ*, we are deleting more edges, so the size of the edge set decreases. Thus, the binary network satisfies the monotonic subset property

$$X_{\epsilon_{0}}⊃X_{\epsilon_{1}}⊃X_{\epsilon_{2}}⊃\cdots$$

for any *ϵ*_0_ ≤ *ϵ*_1_ ≤ *ϵ*_2_ ⋯. Equivalently, we also have

$$X_{\epsilon_{0}}^{c}\subset X_{\epsilon_{1}}^{c}\subset X_{\epsilon_{2}}^{c}\subset \cdots .$$

The sequence of such nested multiscale graphs is defined as the graph filtration (Lee et al., 2011b, 1). Note that 𝒳_0_ is the complete graph and 𝒳_∞_ is the node set *V*. For a graph with *p* nodes, the maximum number of edges is (*p*^2^ − *p*)/2, which is obtained in a complete graph. If we order the edge weights in increasing order, we have the sorted edge weights:

$$0=w_{\left(0\right)}<\underset{j,k}{\min}w_{jk}=w_{\left(1\right)}<w_{\left(2\right)}<\cdots <w_{\left(q\right)}=\underset{j,k}{\max}w_{jk},$$

where *q* ≤ (*p*^2^ − *p*)/2. The subscript _( )_ denotes the order statistic. Hence, we simply construct the graph filtration at the edge weights

$$X_{0}⊃X_{w_{\left(1\right)}}⊃X_{w_{\left(2\right)}}⊃\cdots ⊃X_{w_{\left(q\right)}}.$$ (2)

The condition of having unique edge weights is not restrictive in practice. Assuming edge weights to follow some continuous distribution, the probability of any two edges being equal is zero. The finiteness and uniqueness of the filtration levels over finite graphs are intuitively clear by themselves and are implicitly assumed in software packages such as javaPlex (Adams, Tausz, & Vejdemo-Johansson, 2014).

## BETTI NUMBERS

In persistent homology, the *k*-th Betti number is often referred to as the number of *k*-dimensional holes (Lee et al., 2014, 1; Petri et al., 2014; Sizemore et al., 2018). In network setting, the 0-th Betti number is the number of connected components and the 1st Betti number is the number of cycles. During graph filtration, we can show that *β*_0_ and *β*_1_ monotonically change. Although it is not true in general (Bobrowski & Kahle, 2014), on the graph filtration (2), *β*_0_ and *β*_1_ numbers have very stable monotonic increases and decreases respectively.

Theorem 1 In a graph, Betti numbers *β*_0_ and *β*_1_ are monotone over graph filtration on edge weights.

*Proof*. Under graph filtration (2), the edges are deleted one at a time. Since an edge has only two end points, the deletion of the edge disconnects the graph into at most two. Thus, the number of connected components (*β*_0_) always increases, and the increase is at most by one. The Euler characteristic *χ* of the graph is given by (Adler, Bobrowski, Borman, Subag, & Weinberger, 2010)

$$\chi =\beta_{0}-\beta_{1}=p-q,$$

where *p* and *q* are the number of nodes and edges respectively. Thus,

$$\beta_{1}=\beta_{0}-p+q.$$

Note *p* is fixed over the filtration but *q* is decreasing by one while *β*_0_ increases at most by one. Hence, *β*_1_ always decreases and the decrease is at most by one.

Theorem 1 is related to the incremental Betti number computation over a simplical complex (Boissonnat & Teillaud, 2006). Once we compute *β*_0_ number, *β*_1_ number is simply given by *β*_0_ − *p* + *q* without additional computation. For the computation of *β*_0_, it is not necessary to perform graph filtration for infinitely many possible filtration values. The maximum possible number of filtration level needed for computing *β*_0_ is one plus the number of unique edge weights. In the case of trees, *β*_0_ computation is exactly given.

Theorem 2 For tree 𝒯 = (*V*, *w*) with *p* ≥ 2 nodes and unique positive edge weights

$$w_{\left(1\right)}<w_{\left(2\right)}<\cdots <w_{\left(p-1\right)},$$

the zeroth Betti number *β*_0_ over graph filtration (2) is given by

$$\beta_{0}(T_{0})=1,\;\beta_{0}(T_{w_{\left(1\right)}})=2,\;\cdots ,\;\beta_{0}(T_{w_{\left(p-1\right)}})=p.$$

The proof is given in Chung et al. (2015). Note a tree with *p* nodes has *p* − 1 edges. For a graph that is not possible, it may not be possible to analytically represent *β*_0_ over a filtration like Theorem 2. In general, *β*_0_ can be numerically computed using the single linkage dendrogram (SLD) (Lee et al., 2012), the Dulmage-Mendelsohn decomposition (Chung, Adluru, Dalton, Alexander, & Davidson, 2011; Pothen & Fan, 1990), or the simplical complex method (Carlsson & Memoli, 2008; de Silva & Ghrist, 2007; Edelsbrunner, Letscher, & Zomorodian, 2002). In this study, we computed *β*_0_ over filtration by using the Dulmage-Mendelsohn decomposition.

## SINGLE LINKAGE CLUSTERING

The *β*_0_ computation is related to single linkage clustering and dendrogram construction (Carlsson, 2009; Carlsson, De Silva, & Morozov, 2009; Carlsson, Singh, & Zomorodian, 2009b; Chowdhury & Mémoli, 2016; Khalid, Kim, Chung, Ye, & Jeon, 2014). In single linkage clustering, the single linkage distance (SLD) *s*_*ij*_ between the closest nodes in the two disjoint connected components **R**_1_ and **R**_2_ is given by

$$s_{ij}=\underset{l\in R_{1},k\in R_{2}}{\min}w_{lk}.$$

In this study, the square-root of 1 correlation is used as edge weight *w*_*kl*_.

**Figure 1.**
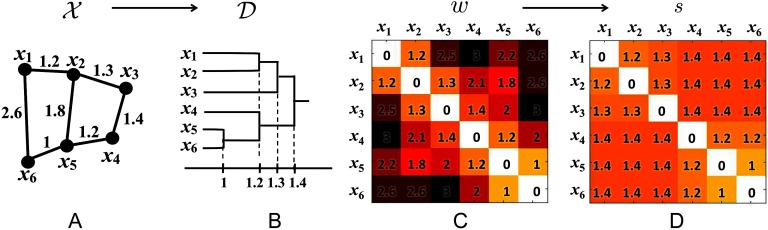
(A) Toy network, (B) its dendrogram, (C) the distance matrix *w* based on Euclidean distance, and (D) the single linkage matrix *S*. In the case of dendrogram construction, the graph filtration is done by connecting nodes over increasing edge weights.

Every edge connecting a node in **R**_1_ to a node in **R**_2_ has the same SLD. The SLD is then used to construct the single linkage matrix (SLM) *S* = (*s*_*ij*_) (Figure 1). SLM shows how connected components are merged locally and can be used in constructing a dendrogram over filtration. If the single linkage distance *s*_*ij*_ is larger than the current filtration value *ϵ*_*k*_ but smaller than the next filtration value *ϵ*_*k*+1_, that is, *ϵ*_*k*_ ≤ *s*_*ij*_ < *ϵ*_*k*+1_. Then components **R**_1_ and **R**_2_ will be connected at the next filtration value *ϵ*_*k*+1_. The sequence of how components are merged during the graph filtration is identical to the sequence of the merging in the dendrogram construction (Lee et al., 2012). By tracing how each of the connected components are merged, we can compute *β*_0_. In the single linkage clustering, instead of deleting edges, we are connecting nodes over increasing edge weights.

SLM is an *ultrametric*, which is a metric space satisfying the stronger triangle inequality *s*_*ij*_ ≤ max(*s*_*ik*_, *s*_*kj*_) (Carlsson & Mémoli, 2010). Thus the dendrogram can be represented as an ultrametric space 𝒟 = (*V*, *S*), which is again a metric space. In persistent homology, the Gromov-Hausdorff (GH) distance has been mainly used in quantifying the dendrogram shape differences (Carlsson & Mémoli, 2010; Chung et al., 2017a; Lee et al., 2011b, 1). The GH-distance between dendrograms 𝒟^1^ = (*V*, *S*^1^) and 𝒟^2^ = (*V*, *S*^2^) with SLM *S*^1^ = ($s_{ij}^{1}$) and *S*^2^ = ($s_{ij}^{2}$) is given by

$$D_{GH}(D^{1},D^{2})=\frac{1}{2}\underset{\forall i,j}{\max}|s_{ij}^{1}-s_{ij}^{2}|.$$

For the statistical inference on GH-distance, resampling techniques such as jackknife or permutation tests are often used ((Lee et al., 2012), 1). In this study, we will use the permutation test.

## BOTTLENECK DISTANCE

The bottleneck distance is perhaps the most often used distance in persistent homology, although it is *rarely* used for brain networks. In persistent homology, the topology of underlying data can be represented by the birth and death of topological features, such as the number of connected components or cycles (Carlsson, Ishkhanov, De Silva, & Zomorodian, 2008). During the filtration, these topological features appear and disappear. If a topological feature appears at the threshold *ξ* and disappears at *τ*, it can be encoded into a point, (*ξ*, *τ*) (0 ≤ *ξ* ≤ *τ* < ∞) in ℝ^2^. If *m* number of connected components or cycles appear during the filtration of a network 𝒳 = (*V*, *w*), the homology group can be represented by a point set

$$P(X)=\left\{\left(\xi_{1},\tau_{1}),\ldots ,(\xi_{m},\tau_{m}\right)\right\}.$$

This scatter plot is called a persistence diagram (PD) (Cohen-Steiner, Edelsbrunner, & Harer, 2007).

Given two networks 𝒳^1^ = (*V*^1^, *w*^1^) with *m* features and 𝒳^2^ = (*V*^2^, *w*^2^) with *n* features, PDs

$$P(X^{1})=\left\{\left(\xi_{1}^{1},\tau_{1}^{1}),\cdots ,(\xi_{m}^{1},\tau_{m}^{1}\right)\right\}$$

and

$$P(X^{2})=\left\{\left(\xi_{1}^{2},\tau_{1}^{2}),\cdots ,(\xi_{n}^{2},\tau_{n}^{2}\right)\right\}$$

are obtained through the filtration (Lee et al., 2012). The bottleneck distance between the networks is defined as the bottleneck distance of the corresponding PDs (Cohen-Steiner et al., 2007):

$$D_{B}(P(X^{1}),P(X^{2}))=\underset{\gamma }{\inf}\underset{1\leq i\leq m}{\sup}∥t_{i}^{1}-\gamma (t_{i}^{1}){∥}_{\infty },$$ (3)

where $t_{i}^{1}$ = ($\xi_{i}^{1}$, $\tau_{i}^{1}$) ∈ 𝒫(𝒳^1^) and *γ* is a bijection from 𝒫(𝒳^1^) to 𝒫(𝒳^2^). The infimum is taken over all possible bijections. If $t_{j}^{2}$ = ($\xi_{j}^{2}$, $\tau_{j}^{2}$) = *γ*($t_{i}^{1}$) for some *i* and *j*, *L*_∞_-norm is given by

$$∥t_{i}^{1}-\gamma (t_{i}^{1}){∥}_{\infty }=\max(|\xi_{i}^{1}-\xi_{j}^{2}|,|\tau_{i}^{1}-\tau_{j}^{2}|).$$

Note Equation 3 assumes *m* = *n* such that the bijection *γ* exists. Suppose two networks share the same node set, that is, *V*^1^ = *V*^2^, with *p* nodes and the same number of *q* unique edge weights. If the graph filtration is performed on two networks, the number of connected components and cycles that appear and disappear during the filtration is *p* and 1 − *p* + *q*, respectively. Thus, their persistence diagrams always have the same number of points. The bijection *γ* is determined by the bipartite graph matching algorithm (Cohen-Steiner et al., 2007; Edelsbrunner & Harer, 2008).

If *m* ≠ *n*, there is no one-to-one correspondence between two PDs. Then, auxiliary points

$$\left(\frac{\xi_{1}^{1}+\tau_{1}^{1}}{2},\frac{\xi_{1}^{1}+\tau_{1}^{1}}{2}),\cdots ,(\frac{\xi_{m}^{1}+\tau_{m}^{1}}{2},\frac{\xi_{m}^{1}+\tau_{m}^{1}}{2}\right)$$

and

$$\left(\frac{\xi_{1}^{2}+\tau_{1}^{2}}{2},\frac{\xi_{1}^{2}+\tau_{1}^{2}}{2}),\cdots ,(\frac{\xi_{n}^{2}+\tau_{n}^{2}}{2},\frac{\xi_{n}^{2}+\tau_{n}^{2}}{2}\right)$$

that are orthogonal projections to the diagonal line *ξ* = *τ* in 𝒫(𝒳^1^) and 𝒫(𝒳^2^) are added to 𝒫(𝒳^2^) and 𝒫(𝒳^1^), respectively, to make the identical number of points in PDs.

The bottleneck distance does not directly measure the distance between two metric spaces 𝒳^1^ = (*V*^1^, *w*^1^) and 𝒳^2^ = (*V*^2^, *w*^2^), but measures the distance between their corresponding persistence diagrams 𝒫(𝒳^1^) and 𝒫(𝒳^1^). In practice, the bottleneck distance has been often used since it is a lower bound on the GH-distance and it is easier to compute (Chazal et al., 2009). Since the brain regions that form the network nodes are matched across the networks through predefined parcellations in brain network studies, the GH-distance can be computed easily. Thus, in this study, we will only use the GH-distance and not show the result of the bottleneck distance in the simulation study.

## PERMUTATION TEST ON NETWORK DISTANCES

Statistical inference on network distances can be done using resampling techniques such as the permutation test (Chung et al., 2013; Efron, 1982; Lee et al., 2012). The permutation test is perhaps the most widely used nonparametric test procedure in the sciences (Chung et al., 2017b; Nichols & Holmes, 2002; Thompson, Cannon, Narr, van Erp, Poutanen, Huttunen, Lonnqvist, Standertskjold-Nordenstam, Kaprio, & Khaledy, 2001; Zalesky, Fornito, Harding, Cocchi, Yücel, Pantelis, & Bullmore, 2010). It is known as the exact test in brain imaging since the distribution of the test statistic under the null hypothesis can be exactly computed if we can calculate all possible values of the test statistic under every possible permutation.

Here we explain the permutation test procedure that was used for network distances. The usual setting in brain imaging applications is a two-sample comparison. Suppose there are *m* measurement in Group 1 on node set *V* of size *p*. Denote the data matrix as $X_{m\times p}^{1}$. The edge weights of Group 1 are given by *f*(**x**^1^) for some function *f* and the metric space is given by 𝒳^1^ = (*V*, *f*(**X**^1^)). Suppose there are *n* measurement in Group 2 on the identical node set *V*. Denote data matrix as $X_{n\times p}^{2}$ and the corresponding metric space as 𝒳^1^ = (*V*, *f*(**X**^1^)). We test the statistical significance of network distance *D*(𝒳^1^, 𝒳^2^) under the null hypothesis *H*_0_:

$$H_{0}:D(X^{1},X^{2})=0\;\text{vs.}\;H_{1}:D(X^{1},X^{2})>0.$$

The permutation test is done as follows. Under *H*_0_, one can concatenate the data matrices

$$X=(x_{ij})={\left(\begin{array}{c} X^{1}\\ X^{2} \end{array}\right)}_{(m+n)\times p}$$

and then permute the indices of the row vectors of **X** in the symmetric group of degree *m* + *n*, that is, *S*_*m*+*n*_ (Kondor, Howard, & Jebara, 2007). Denote the *i*-th permuted data matrix as **X**_*σ*(*i*)_ = (*x*_*σ*(*i*),*j*_), where *σ* ∈ *S*_*m*+*n*_. Then we split **X**_*σ*(*i*)_ into submatrices such that

$$X_{\sigma (i)}=\left(\begin{array}{c} X_{\sigma (i)}^{1}\\ X_{\sigma (i)}^{2} \end{array}\right),$$

where $X_{\sigma (i)}^{1}$ and $X_{\sigma (i)}^{2}$ are of sizes *m* × *p* and *n* × *p* respectively. Let $X_{\sigma (i)}^{1}$ = (*V*, *f*($X_{\sigma (i)}^{1}$)) and $X_{\sigma (i)}^{2}$ = (*V*, *f*($X_{\sigma (i)}^{2}$)) be weighted networks where the rows of the data matrices are permuted across the groups. Then we have distance *D*($X_{\sigma (i)}^{1}$, $X_{\sigma (i)}^{2}$) for each permutation. The fraction of permutations *D*($X_{\sigma (i)}^{1}$, $X_{\sigma (i)}^{2}$) that is larger than *D*(𝒳^1^, 𝒳^2^) gives the estimate for the *p* value.

Unfortunately, generating every possible permutation for whole images is still extremely time consuming even for a modest sample size. The number of permutations exponentially increases, and it is impractical to generate every possible permutation. In the permutation test, only a small fraction of possible permutations are generated, and the statistical significance is computed approximately. In most studies, on the order of 1% of total permutations were often used, mainly due to the computational bottleneck of generating permutations (Thompson et al., 2001; Zalesky et al., 2010). In Zalesky et al. (2010), 5,000 permutations out of possible 2712 = 17,383,860 permutations (2.9%) were used. In Thompson et al. (2001), 1 million permutations out of 4020 possible permutations (0.07%) were generated using a super computer. In our study, we have 131 MZ and 77 DZ twins. The possible number of permutations is 20877. This is a number so large, we cannot exactly represent it in computing systems such as MATLAB and R. Even the 1% of 20877 is about 1.96 × 10^56^, which is still astronomically large and beyond the computing capability of the most computers. On the other hand, the proposed KS-distance method computes for all possible permutations combinatorially and completely bypasses the computational bottleneck. There is no computational cost involved in the KS-distance and the computation is done in a few seconds. Furthermore, the method computes *p* values exactly and it is not approximate.

## KOLMOGOROV-SMIRNOV DISTANCE

Recently, the Kolmogorov-Smirnov (KS) distance has been successfully applied in quantifying the change of *β*_0_ number over graph filtration as a way to quantify brain networks without thresholding (Chung et al., 2017a, 2017b). The main advantage of the method is that it avoids using the computationally costly and time consuming permutation test for large-scale networks. In this paper, we show how to apply KS-distance in quantifying the change of the *β*_1_ number over graph filtration as well.

In this study, the square root of 1 correlation is used as edge weights. Given two networks 𝒳^1^ = (*V*, *w*^1^) and 𝒳^2^ = (*V*, *w*^2^), KS distances between 𝒳^1^ and 𝒳^2^ for Betti numbers *β*_0_ and *β*_*j*_ are defined as (Chung et al., 2013; Lee et al., 2017):

$$D_{KS}(X^{1},X^{2})=\underset{\epsilon \geq 0}{\sup}|\beta_{j}(X_{\epsilon }^{1})-\beta_{j}(X_{\epsilon }^{2})|,$$

where *β*_*j*_($X_{\epsilon }^{i}$) is the *j*-th Betti number for binary network $X_{\epsilon }^{i}$. The distance *D*_*KS*_ can be discretely approximated using the finite number of filtrations:

$$D_{q}=\underset{1\leq i\leq q}{\sup}|\beta_{j}(X_{\epsilon_{i}}^{1})-\beta_{j}(X_{\epsilon_{i}}^{2})|.$$

If we choose enough of *q* such that *ϵ*_*j*_ are all the sorted edge weights, then

$$D_{KS}(X^{1},X^{2})=D_{q}$$

(Chung et al., 2017b). This is possible since there are only up to *p*(*p* − 1)/2 number of unique edges in a graph with *p* nodes and the monotone function increases discretely but *not continuously*. In practice, *ϵ*_*j*_ may be chosen uniformly or a divide-and-conquer strategy can be used to adaptively grid the filtration values. Then the probability distribution of *D*_*q*_ can be computed exactly by combinatorial means.

Theorem 3 P(Dq≥d)=1−Aq,q2qq,

where *A*_*u*,*v*_ satisfies *A*_*u*,*v*_ = *A*_*u*−1,*v*_ + *A*_*u*,*v*−1_ with the boundary condition *A*_0,*v*_ = *A*_*u*,0_ = 1 within band |*u* − *v*| < *d* and initial condition *A*_0,0_ = 0 for *u*, *v* ≥ 1.

The proof is given in Chung et al. (2017b).

**Figure 2.**
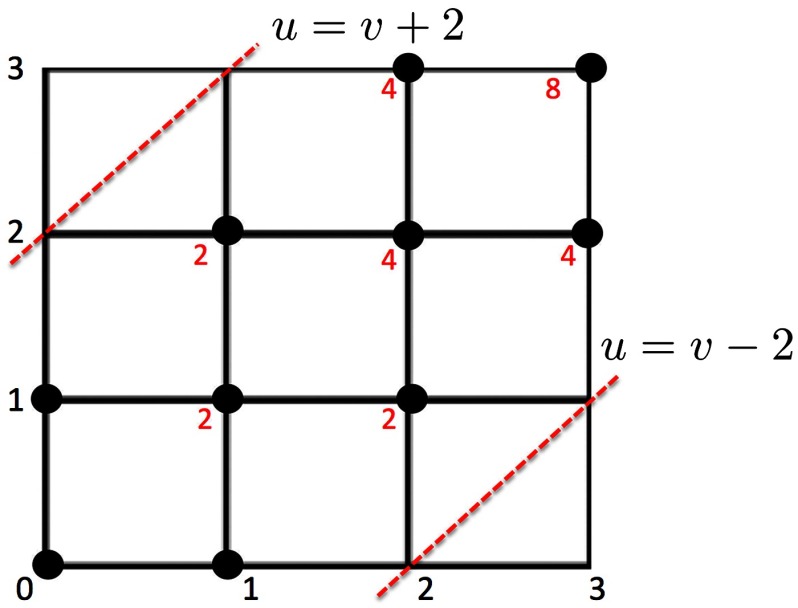
In this example, *A*_*u*,*v*_ is computed within the boundary (dotted red line) from (0, 0) to (3, 3).

Example 1 *P*(*D*_3_ ≥ 2) is computed sequentially as follows (Figure 2). We start with the bottom left corner *A*_0,0_ = 0 and move right or up toward the upper corner

$$\begin{array}{ccc} A_{1,0} & = & 1,A_{0,1}=1\\ \to A_{1,1} & = & A_{1,0}+A_{0,1}\\ \to \cdots & = & \cdots \\ \to A_{3,3} & = & A_{3,2}+A_{2,3}=8. \end{array}$$

The probability is then *P*(*D*_3_ ≥ 2) = 1 − 8/63 = 0.6. The computational complexity of the combinatorial inference is 𝒪(*q* log *q*) for sorting and 𝒪(*q*^2^) for computing *A*_*q*,*q*_ in the grid while the permutation test requires exponential run time.

When *q* is too large, it may not be possible to represent and compute 2qq in all the digits. For large *q*, use the asymptotic probability distribution *D*_*q*_ given by Chung et al. (2017b):

$$\underset{q\to \infty }{\lim}(D_{q}/\sqrt{2q}\geq d)=2\overset{\infty }{\underset{i=1}{\sum }}{\left(-1\right)}^{i-1}e^{-2i^{2}d^{2}}.$$ (4)

The *p* value of the test statistic under the null is then computed as

$$p\;\text{value}=2e^{-d_{o}^{2}}-2e^{-8d_{o}^{2}}+2e^{-18d_{o}^{2}}\cdots ,$$

where the observed value *d*_*o*_ is the least integer greater than *D*_*q*_/$\sqrt{2q}$ in the data.

## COMPARISONS

**Figure 3.**
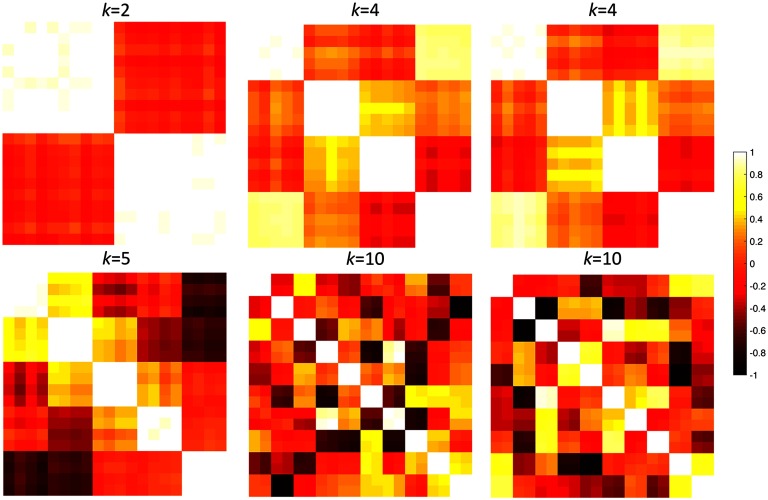
Randomly simulated correlation matrices with different topological structures with *k* = 2, 4, 5, 10. For *k* = 4, 10, two different randomly generated networks are shown.

Six network distances (*L*_1_, *L*_2_, *L*_∞_, GH and KS on *β*_0_ and *β*_1_) were compared in simulation studies. For the review of various brain network distances, refer to Chung et al. (2017a). We also used the popular Q-modularity function for community detection in graph theory (Girvan & Newman, 2002; Meunier et al., 2009; Newman et al., 2006). The difference in Q-modularity functions was used as the distance measure. The simulations below were independently performed 100 times. We used *p* = 20,100,500 nodes and *n* = 5 images in each group, which made it possible for permutations to be exactly 5+55 = 252 (Figure 3). The small number of permutations enables us to compare the performance of distances exactly. Through the simulations, *σ* = 0.1 was universally used as network variability.

The data vector **x**_*i*_ at node *i* was simulated as identical and independently distributed multivariate normal across *i*, that is, **x**_*i*_ ∼ *N*(0, *I*_*n*_) with *n* by *n* identity matrix *I*_*n*_ as the covariance matrix. This gives the correlation matrix *C*^1^ = ($c_{ij}^{1}$) = (*corr*(**x**_*i*_, **x**_*j*_)). The edge weights were given by $\sqrt{1-c_{ij}^{1}}$. The data vector **y**_*i*_ at node *i* that produced node dependency was simulated by adding additional dependency to **x**_*i*_ through a hierarchical linear model or mixed-effect model (Pinehiro & Bates, 2002; Snijders, Spreen, & Zwaagstra, 1995). This is a standard simulation technique for introducing dependency structures in random simulations. The hierarchical linear model enables us to explicitly model the data vector at each node and simulate the amount of dependency between nodes, providing detailed control over the topological structures in the correlation matrices. Data vector **y**_*i*_ at node *i* will be simulated using **x**_*i*_ as follows.

$$\begin{array}{ccc} y_{1},\cdots ,y_{c} & = & x_{1}+N(0,\sigma^{2}I_{n})\\ y_{c+1},\cdots ,y_{2c} & = & x_{c+1}+N(0,\sigma^{2}I_{n})\\ & & \vdots \\ y_{c(k-1)+1},\cdots ,y_{ck} & = & x_{c(k-1)+1}+N(0,\sigma^{2}I_{n}) \end{array}$$

This introduces a topological structure of connectedness through statistical dependency. Although we did not try here, a far more complex dependency structure is also possible. In our simulation *c* = *p*/*k* = 10, 5, 4, 2 and *k* = *p*/*c* = 2, 4, 5, 10 are used (Figure 3). Subsequently, we have the correlation matrix *C*^2^ = ($c_{ij}^{2}$) = (*corr*(**y**_*i*_, **y**_*j*_)) and the subsequent edge weights $\sqrt{1-c_{ij}^{2}}$.

### No Network Difference

It was expected there was no network difference between networks generated using the same parameters and initial data vectors **x**_*i*_ in the above model. For example, Figure 3 shows two simulated networks generated with the same parameters *k* = 4, 10. We compared networks with the same parameter *k*: 4 vs. 4, 5 vs. 5 and 10 vs. 10. It is expected we should not able to detect the network differences. The performance results were given in terms of the false positive error rate computed as the fraction of simulations that gave *p* value below 0.05 (Table 1). For all the distances except KS-distance, the permutation test was used. Since there were five samples in each group, the total number of permutations was 105 = 272, making the permutation test exact and the comparisons accurate. All the distances performed very well including Q-modularity. KS-distance was overly sensitive and was producing up to 7% false positives. However, for 0.05 level test, it is expected that there is 5% chance of producing false positives. Thus, KS-distance is producing only 2% above the expected error rate.

**Table 1.** The *p* = 20 nodes simulation results given in terms of false positive and negative error rates.

*p* = 20	*L*_1_	*L*_2_	*L*_∞_	GH	KS (*β*_0_)	KS (*β*_1_)	Q
4 vs. 4	0.00	0.00	0.00	0.00	0.04	0.01	0.05
5 vs. 5	0.00	0.00	0.00	0.00	0.07	0.01	0.06
10 vs. 10	0.00	0.00	0.00	0.00	0.00	0.00	0.04

4 vs. 5	0.63	0.40	0.33	0.15	0.27	0.06	0.9
2 vs. 4	0.71	0.48	0.42	0.53	0.18	0.00	0.95
5 vs. 10	0.94	0.80	0.78	0.72	0.44	0.24	0.96

The *p* = 20 simutation might be too small a network to extract topologically distinct features that are used in topological distances. Thus, we increased the number of nodes to *p* = 100 (Table 2). All the network distances except KS-distances performed reasonably well. KS-distances seem to be overly sensitive to slight topological change in large topological structures that were present in *k* = 2, 4, 5 cases. As *k* increases, KS-distances seem to perform reasonably well.

**Table 2.** The *p* = 100 nodes simulation results given in terms of false positive and negative error rates.

*p* = 100	*L*_1_	*L*_2_	*L*_∞_	GH	KS (*β*_0_)	KS (*β*_1_)	Q
4 vs. 4	0.00	0.00	0.00	0.00	0.26	0.54	0.03
5 vs. 5	0.00	0.00	0.00	0.00	0.14	0.43	0.05
10 vs. 10	0.00	0.00	0.00	0.00	0.05	0.05	0.05

4 vs. 5	0.51	0.37	0.35	0.16	0.11	0.00	0.93
2 vs. 4	0.66	0.45	0.57	0.61	0.03	0.00	0.91
5 vs. 10	0.94	0.86	0.79	0.72	0.11	0.00	0.98

### Network Differences

We generated networks with parameter *k* = 2, 4, 5, 10 with *p* = 20 nodes simulation (Figure 3). Since topological structures were different, the distances are expected to differentiate the networks. The performance results were given in terms of the false negative error rate computed as the fraction of simulations that give *p* value above 0.05 (Table 1). All the distances including Q-modularity performed badly, although KS-distance performed the best. Since graph theory features are *not explicitly* designed to measure network distances, they do not usually perform well when there are large topological differences.

We increased the number of nodes to *p* = 100. All the network distances including Q-modularity were still performing badly except KS-distances (Table 2). KS-distance on the number of cycles seems to be the best network distance to use when there are network topology differences, although it has tendency to produce false positives when there is no difference.

In terms of computation, distance methods based on the permutation test took about 950 seconds (16 minutes) for 100 nodes, while the KS-like test procedure only took about 20 seconds in a computer. The results given in Tables 1–3 may slightly change if different random networks are generated. We also performed the simulation study on the 500 nodes to see the effect of increased network sizes (Table 3). The proposed KS-distance on both *β*_0_ and *β*_1_ are not necessarily performing well in the case of no network differences. Again the KS-distance is too sensitive and detecting minute network differences. On the other hand, in the case of actual network differences, the KS-distances are performing exceptionally well compared with other network differences.

**Table 3.** The *p* = 500 nodes simulation results given in terms of false positive and negative error rates.

*p* = 500	*L*_1_	*L*_2_	*L*_∞_	GH	KS (*β*_0_)	KS (*β*_1_)	Q
4 vs. 4	0.04	0.05	0.06	0.08	0.20	0.26	0.02
5 vs. 5	0.00	0.00	0.00	0.00	0.13	0.20	0.02
10 vs. 10	0.00	0.00	0.00	0.00	0.06	0.18	0.05

4 vs. 5	0.20	0.20	0.20	0.20	0.11	0.00	0.20
2 vs. 4	0.14	0.11	0.14	0.12	0.00	0.00	0.17
5 vs. 10	0.20	0.18	0.19	0.16	0.00	0.00	0.20

## APPLICATION

As an application, we show how to apply KS-distances in understanding heritability of brain networks. Because of their unique relationship, twin imaging studies allow researchers to examine genetic and environmental influences easily *in vivo* (Blokland, McMahon, Thompson, Martin, de Zubicaray, & Wright, 2011; Chiang, McMahon, de Zubicaray, Martin, Hickie, Toga, Wright, & Thompson, 2011; Glahn, Winkler, Kochunov, Almasy, Duggirala, Carless, Curran, Olvera, Laird, Smith, Beckmann, Fox, & Blangero, 2010; McKay, Knowles, Winkler, Sprooten, Kochunov, Olvera, Curran, Kent Jr., Carless, Göring, Dyer, Duggirala, Almasy, Fox, Blangero, & Glahn, 2014; Smit, Stam, Posthuma, Boomsma, & De Geus, 2008). Monozygotic (MZ) twins share 100% of genes, whereas dizygotic (DZ) twins share 50% of genes (Chung et al., 2017b). The difference between MZ and DZ twins measures the degree of genetic and environmental influence. Twin imaging studies are very useful for understanding the extent to which brain networks are influenced by genetic factors. This information can then be later used to develop better ways to prevent and treat disorders and maladaptive behaviors.

### Dataset and Image Preprocessing

We used the resting-state fMRI of 271 twin pairs from the Human Connectome Project (Van Essen, Ugurbil, Auerbach, Barch, Behrens, Bucholz, Chang, Chen, Corbetta, & Curtiss, 2012). Out of a total 271 twin pairs, we only used genetically confirmed 131 MZ twin pairs (age 29.3 ± 3.3 years, 56M/75F) and 77 same-sex DZ twin pairs (age 29.1 ± 3.5 years, 30M/47F) in this study. Since the discrepancy between self-reported and genotype-verified zygosity was fairly high at 13% of all the available data, 19 MZ and 19 DZ twin pairs that do not have genotyping were excluded. We additionally excluded 35 twin pairs with missing fMRI data.

fMRI were collected on a customized Siemens 3T Connectome Skyra scanner, using a gradient-echo-planar imaging (EPI) sequence with multiband factor = 8, TR = 720 ms, TE = 33.1 ms, flip angle = 52°, 104 × 90 (RO×PE) matrix size, 72 slices, and 2-mm isotropic voxels; 1,200 volumes were obtained over a 14 min, 33 sec scanning session. fMRI data has undergone spatial and temporal preprocessing including motion and physiological noise removal (Smith et al., 2013). Using the resting-state fMRI, we employed the Automated Anatomical Labeling (AAL) brain template to parcellate the brain volume into 116 regions (Tzourio-Mazoyer, Landeau, Papathanassiou, Crivello, Etard, Delcroix, Mazoyer, & Joliot, 2002). The fMRI were then averaged across voxels in each brain region for each subject. The averaged fMRI signal in each parcellation was then temporally smoothed using the cosine series representation as follows (Chung, Adluru, Lee, Lazar, Lainhart, & Alexander, 2010; Gritsenko, Lindquist, Kirk, & Chung, 2018).

Given fMRI time series at the *i*-th parcellation *ζ*_*i*_(*t*) at time *t*, we scaled it to fit to unit interval [0, 1]. Then subtracted its mean over time $\int_{0}^{1}$ *ζ*_*i*_(*t*) *dt*. Then the resulting scaled and translated time series was represented as

$$\zeta_{i}(t)=\overset{k}{\underset{l=0}{\sum }}c_{li}\psi_{l}(t),\;t\in [0,1],$$

where *ψ*_0_(*t*) = 1, *ψ*_*l*_(*t*) = $\sqrt{2}$ cos(*lπt*) were cosine basis functions and *c*_*li*_ were coefficients estimated in the least squares fashion. For our study, *k* = 119 was used such that fMRI were compressed into 10% of the original data size; *k* = 119 expansion increased the signal-to-noise ratio (SNR) as measured by the ratio of variabilities by 81% in average over all 116 brain regions and 416 subjects, that is, SNR = 1.81. The resulting real-valued Fourier coefficient vector **c**_*i*_ = (*c*_0*i*_, *c*_1*i*_, ⋯, *c*_*ki*_) was then used to represent the fMRI in each parcellation as 120 features in the spectral domain.

### Twin Correlations

The subject level connectivity matrix *C* = (*c*_*ij*_) was computed by correlating 120 features in the spectral domain. Between *i*- and *j*-th parcellations, the connectivity was measured by correlating **c**_*i*_ and **c**_*j*_ over 120 features, that is, *c*_*ij*_ = *corr*(**c**_*i*_, **c**_*j*_). From the individual correlation matrices *C*, we computed pairwise twin correlations in each group at the edge level. The resulting group level twin correlations matrices *C*_*MZ*_ = ($c_{ij}^{MZ}$) and *C*_*DZ*_ = ($c_{ij}^{DZ}$) are nonsymmetric cross-correlation matrices. Since there is no preference in the order of twins, we symmetrize them by

$$C_{MZ}\leftarrow (C_{MZ}+C_{MZ}^{⊤})/2$$

and

$$C_{DZ}\leftarrow (C_{DZ}+C_{DZ}^{⊤})/2.$$

Then we are interested in knowing the extent of the genetic influence on resting-state functional brain network and its statistical significance. For this, we use the widely used ACE genetic model (Falconer & Mackay, 1995) that mainly uses heritability index (HI), which determines the amount of variation (in terms of percentage) due to genetic influence in a population. HI is often estimated using Falconer’s formula (Falconer & Mackay, 1995) as a baseline. MZ twins share 100% of genes, whereas DZ twins share 50% of genes. Thus, the additive genetic factor *A*, the common environmental factor *C* for each twin type are related as

$$corr(c_{ij}^{MZ})=A+C,$$ (5)

$$corr(c_{ij}^{DZ})=A/2+C,$$ (6)

where *corr*($c_{ij}^{MZ}$) and *corr*($c_{ij}^{DZ}$) are the pairwise correlation within MZ and same-sex DZ twins at edge between *i* and *j*. Solving Equation 5 and Equation 6, we obtain the additive genetic factor, that is, HI given by

$$\text{HI}=2(C_{MZ}-C_{DZ}).$$

The network differences between MZ and DZ twins are considered as mainly contributed to heritability and can be used to determine the statistical significance of HI (Chung et al., 2017, 2018). The KS-distance was computed by taking 1 − *C*_*MZ*_ and 1 − *C*_*DZ*_ as edge weights.

In most brain imaging studies, 5,000–1,000,000 permutations are often used, which puts the total number of generated permutations to usually less than 0.01 to 1% of all possible permutations. In Zalesky et al. (2010), 5,000 permutations are out of a possible 2712 = 17,383,860 permutations (2.9%) used. In Thompson et al. (2001), for instance, 1 million permutations out of 4020 possible permutations (0.07%) were generated using a super computer. In Lee et al. (2017), 5,000 permutations out of a possible 3310 = 92,561,040 permutations (0.005%) were used. Since we have 131 MZ and 77 DZ pairs, the total number of possible permutation is 271131, which is larger than 10^80^. Even if we generate only 0.01% of 10^80^ of all possible permutations, 10^76^ permutations are still too large for most desktop computers. Thus, we choose the KS-distance for measuring the network distance. Although the probability distribution of the KS-distance is actually based on the permutation test but the probability is computed combinatorially, bypassing the need for resampling. KS-distance in our study only took a few seconds to compute the *p* value.

### Results

**Figure 4.**
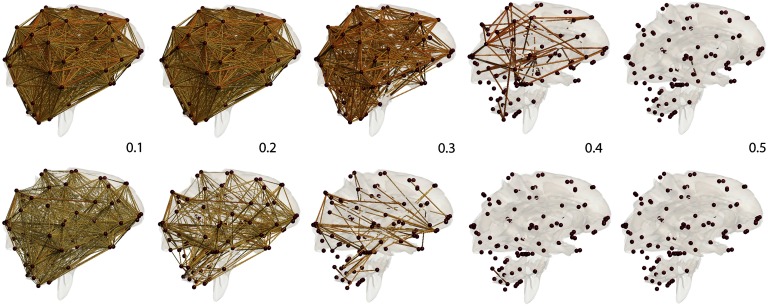
Correlation network filtration thresholded at the indicated correlation values. MZ-twins (top) shows higher correlation connections compared with DZ-twins (bottom). Such connectivity difference is contributed to heritability.

**Figure 5.**
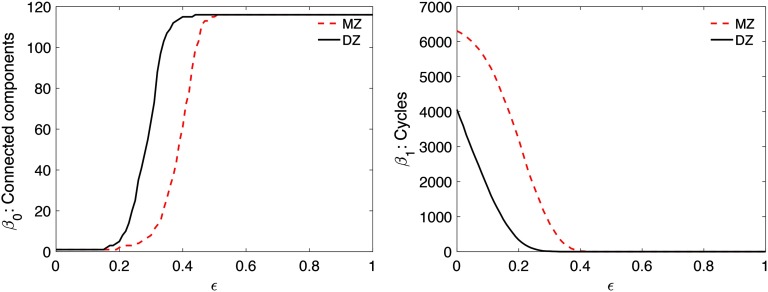
Betti-plots showing Betti numbers over correlation *ϵ* as filtration. MZ twins (top) shows more higher correlation connections and cycles compared with DZ twins (bottom).

We used *β*_0_ and *β*_1_ in computing KS-distances. Let *ϕ* ∘ *C*_*MZ*_ = (*ϕ*($c_{ij}^{MZ}$)) and *ϕ* ∘ *C*_*DZ*_ = (*ϕ*($c_{ij}^{DZ}$)) for some monotone function *ϕ*. Then KS-distance between *C*_*MZ*_ and *C*_*DZ*_ is equivalent to KS-distance between 1 − *C*_*MZ*_ and 1 − *C*_*DZ*_ as well as between *ϕ* ∘ (1 − *C*_*MZ*_) and *ϕ* ∘ (1 − *C*_*DZ*_). Thus, we simply built filtrations over *C*_*MZ*_ and *C*_*DZ*_ and computed KS-distance without using the square-root of 1 - correlation. We used 101 filtration values between 0 and 1 at 0.01 increment (Figure 4). This gives a reasonably accurate estimate of the maximum gap in the *β*_*i*_-plots between the twins (Figure 5). For *β*_0_-plots, the maximum gap is 82, which gives the *p* value smaller than 10^−24^. For *β*_1_-plots, the maximum gap is 3,647, which gives the *p* value smaller than 10^−32^. At the same correlation value, MZ twins are more connected than DZ twins. Also MZ twins have more cycles than DZ twins. Such huge topological differences are contributed to heritability.

**Figure 6.**
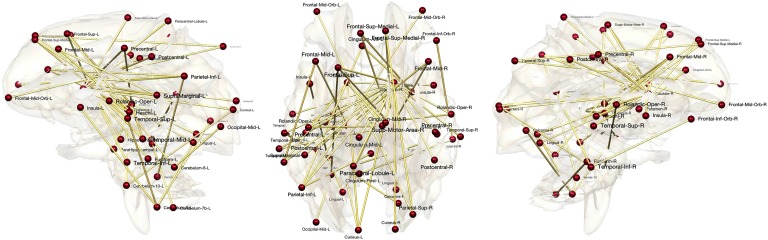
Most highly heritable connections. The connections with 100% heritability are only shown.

Figure 6, which displays the HI index thresholded at 100% heritability, shows MZ twins far more similar compared with DZ twins in many connections, suggesting that genes influence the development of these connections. The most heritable connections include the left frontal gyrus, left and right middle frontal gyri, left superior frontal gyrus, left parahippocampal gyrus, left and right thalami, left and right caudate, and nuclei among many other regions. Most regions overlap with highly heritable regions observed in other twins brain-imaging studies (Fan, Fossella, Sommer, Wu, & Posner, 2003; Glahn et al., 2010; Gritsenko et al., 2018). Moreover, the findings here are somewhat consistent with a previous study on diffusion tensor imaging on twins from our group (Chung, Luo, Adluru, Alexander, Richard, & Goldsmith, 2018a; Chung et al., 2018b), showing that many regions of both resting-state functional and structural connections are heritable at the same time. The left and right caudate nuclei are identified as the most heritable hub nodes in our study.

The MATLAB codes for the simulation study as well as the connectivity matrices *C*_*MZ*_ and *C*_*DZ*_ used in generating results are given at http://www.stat.wisc.edu/∼mchung/TDA.

## DISCUSSION

### The Limitation of KS-distances

Currently KS-distance is applied to Betti numbers *β*_0_ and *β*_1_ separately. It may be possible to construct a new topological distance that uses the combination of both *β*_0_ and *β*_1_ and come up with topologically more sensitive distances. One possible approach is to use the convex combination *α*$D_{KS}^{0}$ + (1 − *α*)$D_{KS}^{1}$, where $D_{KS}^{i}$ is KS-distance for *β*_*i*_ and 0 ≤ *α* ≤ 1. This is beyond the scope of this paper and left as a future study.

### Other Network Distances

The network distances used in this study are not just any other distances but metrics. Since there are almost infinitely many possible similarity measures and distances we can use in networks, the performance of the chosen distance is important in discrimination tasks, which we have shown in simulation studies. The determination of the optimal distance is related to *metric learning*, an area of supervised machine learning in which the goal is to learn from data an optimal similarity function that measures how similar two objects are (Ktena, Parisot, Ferrante, Rajchl, Lee, Glocker, & Rueckert, 2018; Lowe, 1995). This is left as a future study.

### Computational Issues

The total number of permutations in permuting two groups of size *q* each is 2qq ∼ $\frac{4^{q}}{\sqrt{2\pi q}}$. Even for small *q* = 10, more than tens of thousands of permutations are needed for the accurate approximation of the *p* value. The main advantage of KS-distance over all other distance measures is that it avoids numerically performing the permutation test and avoids generating tens of thousands of permutations. Although the probability distribution of the KS-distance is actually based on the permutation test, the probability is computed combinatorially. We believe that it is possible to develop similar theoretical results for other distance measures and come up with a method for avoiding a resampling-based method for statistical inference.

## ACKNOWLEDGMENTS

We thank Yuan Wang of University of South Carolina, Peter Bebunik of University of Florida, Bala Krishnamoorthy of Washington State University, Dustin Pluta of University of California-Irvine, Alex Leow of University of Illinois-Chicago, and Martin Lindquist of Johns Hopkins University for valuable discussions. We also thank Andrey Gritsenko and Gregory Kirk of University of Wisconsin-Madison for logistic support and image preprocessing help.

## AUTHOR CONTRIBUTIONS

Moo Chung: Conceptualization; Data curation; Formal analysis; Funding acquisition; Investigation; Methodology; Project administration; Resources; Software; Supervision; Validation; Visualization; Writing - Original Draft; Writing - Review & Editing. Hyekyoung Lee: Investigation; Methodology; Validation; Visualization; Writing - Original Draft. Alex DiChristofano: Investigation. Hernando Ombao: Writing - Review & Editing. Victor Solo: Conceptualization; Methodology; Writing - Review & Editing.

## FUNDING INFORMATION

Moo Chung, National Institutes of Health (http://dx.doi.org/10.13039/100000002), Award ID: EB022856. Hyekyoung Lee, National Research Foundation of Korea (http://dx.doi.org/10.13039/501100003725), Award ID: NRF-2016R1D1A1B03935463.

## TECHNICAL TERMS

Persistent homology: A topological data analysis technique for computing topological features at different spatial resolutions.
Graph filtration: A collection of nested graphs.
Metric space: A set with a metric defined on the set.
Permutation test: Determines the statistical significance by calculating all possible values of the test statistic under all possible rearrangements of the samples.
Kolmogorov-Smirnov (KS) distance: A distance between the empirical distributions of two samples.
Mixed-effect model: A model with both fixed and random effect terms.
Heritability index: A number between 0 and 1 that measures the amount of genetic contribution.
Betti-plots: Displays the change of Betti numbers over filtration values.
